# *Bifidobacterium longum* 070103 Fermented Milk Improve Glucose and Lipid Metabolism Disorders by Regulating Gut Microbiota in Mice

**DOI:** 10.3390/nu14194050

**Published:** 2022-09-29

**Authors:** Tong Jiang, Ying Li, Longyan Li, Tingting Liang, Mingzhu Du, Lingshuang Yang, Juan Yang, Runshi Yang, Hui Zhao, Moutong Chen, Yu Ding, Jumei Zhang, Juan Wang, Xinqiang Xie, Qingping Wu

**Affiliations:** 1 College of Food Science, South China Agricultural University, Guangzhou 510642, China; 2 Key Laboratory of Agricultural Microbiomics and Precision Application, Ministry of Agriculture and Rural Affairs, Guangdong Provincial Key Laboratory of Microbial Safety and Health, State Key Laboratory of Applied Microbiology Southern China, Institute of Microbiology, Guangdong Academy of Sciences, Guangzhou 510070, China

**Keywords:** *Bifidobacterium longum* fermented milk, glucokinase, glycolipid metabolism disorder, gut microbiota, fecal metabolites

## Abstract

Background: Fermented milk is beneficial for metabolic disorders, while the underlying mechanisms of action remain unclear. This study explored the benefits and underlying mechanisms of *Bifidobacterium longum* 070103 fermented milk (BLFM) in thirteen-week high-fat and high-sugar (HFHS) fed mice using omics techniques. Methods and results: BLFM with activated glucokinase (GK) was screened by a double-enzyme coupling method. After supplementing BLFM with 10 mL/kg BW per day, fasting blood glucose, total cholesterol (TC), low-density lipoprotein cholesterol (LDL-C), and leptin were significantly reduced compared with the HFHS group. Among them, the final body weight (BW), epididymal fat, perirenal fat, and brown fat in BLFM group had better change trends than *Lacticaseibacillus rhamnosus* GG fermented milk (LGGFM) group. The amplicon and metabolomic data analysis identified *Bifibacterium* as a key gut microbiota at regulating glycolipid metabolism. BLFM reverses HFHS-induced reduction in bifidobacteria abundance. Further studies showed that BLFM significantly reduces the content of 3-indoxyl sulofphate associated with intestinal barrier damage. In addition, mice treated with BLFM improved BW, glucose tolerance, insulin resistance, and hepatic steatosis. Conclusion: BLFM consumption attenuates obesity and related symptoms in HFHS-fed mice probably via the modulation of gut microbes and metabolites.

## 1. Introduction

Changes in lifestyle and diet diversification have led to increasing occurrences of abnormal glucose and lipid metabolism and metabolic diseases [1]. Gut microbiota is closely related to the host’s diet and metabolic parameters [2]. Excessive glycolipid intake is related to various tissue and organ dysfunctions, increased intestine permeability, and gut microbiota imbalance [3,4]. In particular, a high-fat diet is significantly related to the ratio of Firmicutes to Bacteroides (F/B) [5]. Mice that were fed *Myrciaria dubia* prevented a decrease in gut microbiota species richness caused by high-fat (HF) diets and high-sugar (HS) diets, reduced the ratio of F/B, and significantly increased *Akkermansia* and *Bifidobacterium* [6] which are negatively correlated with metabolic diseases such as obesity [7]. Rats fed a HF diet and exhibiting metabolic syndrome that were treated with *Bifidobacterium adolescentis* had reduced visceral fat accumulation [8]. Gut microbiota controls metabolic disorders caused by a HF diet by regulating oxidative stress, inflammation, and metabolites [9,10,11]. In vitro selection of probiotic strains to improve glucose and lipid metabolism disorders is mostly based on their redox ability and anti-inflammatory and glycolipid-related drug regulatory targets [12,13,14]. Glucokinase is the first rate-limiting enzyme of glycolysis regulating blood glucose levels in both directions between liver glucose metabolism and insulin secretion [15]. Glucokinase activator SHP289-04 significantly improves hyperglycemia and hyperlipidemia in KKA^y^ mice [16] and glucokinase mediates the expression of insulin receptor substrate 2 to alleviate islet β-cell apoptosis caused by endoplasmic reticulum stress [17]. There are few reports of probiotics as new drug targets. Fermented milk is a suitable carrier for probiotics which contains strains and biologically active compounds produced during fermentation [18]. The beneficial effects of *L. rhamnosus* GG have been extensively studied and have been shown to have good effects, especially in glycolipid metabolism [19,20]. There are many reports on the improvement of glucose and lipid metabolism disorders [21,22]. However, there is a need for systematic research on the improvement of glucose and lipid metabolism parameters and the gut microbiota and its metabolites. This study analyzed the relationship between probiotic fermented milk that activates glucokinase and disorders of glucose and lipid metabolism, intestinal barrier, gut microbiota, and its metabolites, and proposed a plan for improving glucose and lipid metabolism disorders.

## 2. Materials and Methods

### 2.1. Preparation of Probiotic Fermented Milk

A total of 87 lactic acid bacteria (LAB) strains were isolated from food and the feces of healthy people. Human fecal strains are derived from the previous isolation of the research group which received ethical approval [23]. The DNA of the strains were identified using universal primers 27F: 5′-AGAGTTTGATCCTGGCCTCA-3′; 1492R: 5′-GGTTACCTTGTTACGACTT-3′ and Sanger sequencing (Beijing Liuhe Huada Gene Technology Co., Ltd., Beijing, China) and stored in the bacterial strain bank of the Food Safety and Health Team of the Institute of Microbiology, Guangdong Academy of Sciences, China. Bacteria was cultured anaerobically in sterile tryptone-plant peptone-yeast extract broth (TPY) at 37 °C. Skim milk (SM) solution (12% *w*/*v*) was prepared by resuspending the powder (Solarbio, Beijing, China) in deionized water, heat-treated in a sterilization pot (Hirayama HVE-50, Tokyo, Japan) at 105 °C for 15 min, and cooled to room temperature. LAB strains (5% inoculum) were added to SM and cultured anaerobically at 37 °C until the curd was observed for subsequent experiments.

### 2.2. Heterologous Expression and Purification of Glucokinase

The amino acid sequence of human liver glucokinase (GenBank: AAB97682.1) was entrusted to Jinweizhi Company (Suzhou Jinweizhi Biotechnology Co., Ltd., Suzhou, China) for gene synthesis, cloned into pET-28A vector, sequenced (Suzhou Jinweizhi Biotechnology Co., Ltd.), and transformed into BL21 *Escherichia coli*. His-tagged recombinant GK protein was overexpressed by induction with 0.5 mM isopropyl β-D-1-thiogalactopyranoside (IPTG) (Sanggong Bioengineering Co., Ltd., Shanghai, China) at 18 °C for 48 h. Cells were centrifuged at 9000× *g* for 20 min at 4 °C, resuspended in lysis buffer (1 M NaCl, 50 mM phosphate buffer saline, 70 mM imidazole, 1 mg/mL lysozyme, pH = 7.6), and stored at −80 °C. Expression was performed according to a previous method with modifications [24]. Cells were thawed, sonicated on ice using an Ultrasonic cell crusher, and centrifuged at 15,000× *g* for 30 min at 4 °C. The supernatant was filtered with a 0.8 μm filter and placed on a 5 mL HisTrap^TM^ FF protein purification column, and eluted with elution buffer (150 mM NaCl, 50 mM phosphate buffer saline, 200 mM imidazole, pH = 7.2). The eluted proteins were concentrated by ultrafiltration MWCO 30 KDa (Millipore, Burlington, MA, USA). Protein purity was detected by SDS-PAGE.

### 2.3. In Vitro Glucokinase Enzymatic Assays

The curd sample was taken, centrifuged at 4500× *g* for 5 min at 4 °C, and the supernatant obtained. The pH was adjusted to 7.2 with 1 moL/L NaOH solution and centrifuged at 12,000× *g* for 10 min at 4 °C. Then, the supernatant was filtered through a 0.22-μm membrane. The filtrate was stored at −80 °C. Glucokinase activity was measured using the 6-phosphate glucose dehydrogenase coupling method by monitoring the amount of reduced β-nicotinamide adenine dinucleotide (NADH) at A340 nm [25]. By adding fermented milk to the enzyme reaction system to detect the activity of purified recombinant human liver glucokinase, the fermented milk with activation effect was screened for subsequent experiments.

### 2.4. Animal Experiments

Forty SPF male C57/BL6J mice obtained from Guangdong Medical Experimental Animal Center, China were raised in a barrier environment (23.3 °C, 50–60% relative humidity, 12 h/12 h light/dark cycle) with free access to food and water. Mice were adapted for 1 week, then divided into four groups: the control group was fed a basal diet and normal saline levels, the HFHS group was fed a high-fat and high-sugar diet and normal saline; the LGGFG group was fed a HFHS diet and 10 mL/kg BW *Lacticaseibacillus rhamnosus* GG fermented milk per day, while the BLFM group was fed with a HFHS diet containing 10 mL/kg BW *B. longum* 070103 fermented milk per day. The basic feed had 3530 kcal/kg and the energy supply ratio was 20.6% protein, 12% fat, and 67.4% carbohydrate. The HFHS group had 4250 kcal/kg and the energy supply ratio was 16.46% protein, 45.65% fat, and 37.85% carbohydrate. All groups were continuously fed for 13 weeks, and mice BWs were recorded weekly. Mice feces was collected three days before the end of the experiment. At the end of the 13th week, the mice were fasted overnight, the weight recorded, anesthetized with carbon dioxide, the eyeballs removed for blood collection, and the mice were immediately killed by cervical dislocation. The liver, kidney, spleen, perirenal fat, epididymal fat, brown fat, ileum, and colon were dissected, harvested, washed with cold normal saline, and dried on filter paper. The liver, kidney, spleen, perirenal fat, epididymal fat, and brown fat were weighed to calculate the organ index (visceral weight/fasted weight) and fat index (different fat weight/prohibited weight after eating). The tissues of the same sized liver, epididymal fat, brown fat, and colon of each mouse were removed and fixed in 10% formalin for subsequent hematoxylin and eosin (H & E) staining. The remaining tissue was quickly placed on dry ice and stored at −80 °C. All experimental protocols were approved by the Experimental Animal Ethics Committee of the Institute of Microbiology, Guangdong Academy of Sciences (Guangzhou, Guangdong, China; approval number GT-IACUC202104301).

### 2.5. Oral Glucose Tolerance Test (OGTT)

This was performed at the end of the 12th week of the experiment. Mice were fasted overnight and weighed before the test, then gavaged with 20% *w/v* glucose solution at 2 g/kg BW. Blood glucose values were measured at 0, 30, 60, 90, and 120 min after gavage with glucose solution using a blood glucose meter (Yuwell, Nanjing, China), and the area under the curve (AUC) was calculated.

### 2.6. Determination of Serum Metabolites and Intestinal Barrier Index

A blood glucose meter (Yuwell, Shanghai, China) was used on the last day of the experiment to detect fasting blood glucose levels in mice. The collected whole blood was coagulated at room temperature for two hours, centrifuged at 3500× *g* for 15 min and 4 °C, and the supernatant (serum) stored at −80 °C. Serum levels of total cholesterol (TC), thyroglobulin (TG), high density lipoprotein-C (HDL-C), low density lipoprotein-C (LDL-C), aspartate transferase (AST), and alanine transferase (ALT) were measured using a Mindray BS-480 automatic biochemical analyzer (Mindray Co., Ltd., Shenzhen, China), while insulin, leptin, and adiponectin levels were determined using a commercial enzyme-linked immunosorbent assay (ELISA) kit (Dongge Boye Biotechnology Co., Ltd., Beijing, China). The insulin resistance index (HOMA-IR) was expressed as fasting blood glucose (mmol/L) × fasting insulin (mIU/mL)/22.5.

Briefly, 50 mg of colon sample was taken and placed in a cryogenic grinder together with 0.45 mL physiological saline according to a weight-to-volume ratio of 1:9 (g/mL), grinded for 45 s at 60 Hz, 2 times). The supernatant was collected by centrifugation at 13,000× *g* for 15 min at 4 °C. A commercial ELISA kit was used to determine ZO-1 and claudin-1 amounts in colon tissue supernatant (Dongge Boye Biotechnology, Beijing, China).

### 2.7. Histopathological Examination

Dissected mouse liver, epididymal fat, brown fat, and colon tissue were fixed with 10% formalin solution, embedded in paraffin, sliced into 3-mm thick sections using a semi-automatic paraffin microtome (Thermo Fisher Scientific, Waltham, MA, USA), and stained with H & E. Pathological changes were observed with a digital biological microscope (Nanjing Jiangnan Novel Optics Co., Ltd., Nanjing, China) using ScopeImage 9.0 software (Ningbo Yongxin Optics Co., Ltd., Ningbo, China).

### 2.8. Metabolome Analysis of Fecal Metabolites

Fecal metabolites were identified according to a previous method [26] with slight modifications. Fifty mg of mouse feces was placed in a centrifuge tube. Then, 200 μL of extract (2:2:1 methanol:acetonitrile:water) was added, vortexed for 120 s, sonicated at 4 °C for 20 min, incubated at −20 °C for 1 h, and centrifuged at 13,800× *g* for 15 min at 4 °C. The supernatant was evaporated in a vacuum drying oven at room temperature until dryness. The samples were reconstituted with 200 μL of 50% acetonitrile solution, then 20 μL of each sample were mixed as QC samples, and 3 μL of the spiked samples were injected into an Accucore C18 column (100 × 2.1 mm, 2.6 μm; Thermo Fisher Scientific, Waltham, MA, USA) using a Vanquish chromatography system and a Q Exactive™ Plus Hybrid Quadrupole-Orbitrap™ Mass Spectrometer (Thermo Fisher Scientific, Waltham, MA, USA). The mixture was separated using the following elution gradient: 0 min, 2% B; 7 min, 50% B; 19 min, 80% B; 21 min, 98% B; 23 min, 98% B; 23.5 min, 0.2% B where A is either 0.1% formic acid (positive ion mode) or 5 mM acetic acid (negative ion mode), and B is acetonitrile. The samples were scanned in positive and negative ion mode at a flow rate and column temperature of 0.3 mL/min and 35 °C, respectively. The following acquisition parameters were used: resolution, 70,000; automatic gain control (AGC target), 1×10^6^; scanning range, 70–1000 m/z; spray voltage, 3.5 kV. The complete MS/dd-MS2 (topN) from the full mass spectrum was obtained from the five strongest m/z values of the two ionization modes to obtain MS2 spectra of each compound using the following settings: resolution, 17,500; AGC target, 1 × 10^5^; maximum IT, 50 ms; isolation window, 1.5 m/z, normalized collision energy: 20, 40, and 60 eV. The data were analyzed using Compound Discoverer 3.1 software (Thermo Fisher Scientific, Waltham, MA, USA) for baseline correction, peak differentiation, and alignment [27] and the result (CSV file) was exported to Microsoft Excel. MetaboAnalyst 5.0 (https://www.metaboanalyst.ca/MetaboAnalyst/home.xhtml. accessed on 05 September 2022) was used for principal component analysis (PCA), partial least squares discriminant analysis (PLS-DA), and orthogonal partial least squares discriminant analysis (OPLS-DA). Student’s *t*-test was used to calculate the statistical significance (*p* < 0.05) and fold change (FC) between metabolites. Finally, the differential metabolites were screened according to their projection (VIP) score. A VIP > 1.5, *p* < 0.01, and FC > 2 was considered statistically significant. Biochemical databases (HMDB, METLIN, and PUBMED) were used to identify potential markers. The metabolites with significant differences from the control were further analyzed for pathway enrichment analysis and KEGG analysis to determine the influence of consuming probiotic fermented milk on the fecal metabolites of mice on a HFHS diet using the model organism mice as a reference.

### 2.9. Gut Microbiota Analysis

Bacterial genomic DNA was extracted from the colon contents using the E.Z.N.A.^®^ stool DNA kit (Omega Bio-tek, Norcross, GA, USA) and detected by 1% agarose gel electrophoresis. The V3-V4 variable region of the 16S rRNA gene was amplified by polymerase chain reaction (PCR) using primers 338F (5′-ACTCCTACGGGAGGCAGCAG-3′) and 806R (5′-GGACTACHVGGGTWTCTAAT-3′) by an ABI GeneAmp^®^ 9700 PCR thermocycler (ABI, Los Angeles, CA, USA). Cycling conditions of initial denaturation were 95 °C for 3 min, followed by 23 cycles of denaturing at 95 °C for 30 s, annealing at 55 °C for 30 s, extension at 72 °C for 45 s, a single extension at 72 °C for 10 min, and end at 4 °C. PCR products were quantified with a NanoDrop 2000 spectrophotometer (Thermo Fisher Scientific) and separated using 2% agarose gel electrophoresis. Gel products were purified using an AxyPrep DNA gel extraction kit (Axygen Biosciences, Union City, CA, USA), quantified with a Quantus fluorometer (Promega, Madison, WI, USA), and detected by 2% agarose gel electrophoresis. A NEXTflex rapid DNA-Seq kit (Bioo Scientific, Austin, TX, USA) was used for library construction. Sequencing was performed using the Illumina MiSeq PE300 platform (Shanghai Meiji Biomedical Technology Co., Ltd., Shanghai, China).

After demultiplexing, the resulting sequences were merged with FLASH (1.2.11) [28] and quality filtered with fastp (0.19.6) [29]. The high-quality sequences were de-noised using the DADA2 [30] plugin in the Qiime 2 [31] (version 2020.2) pipeline with the recommended parameters to obtain single nucleotide resolution based on error profiles within samples. The sequencing depth reached 97.90% after noise reduction and leveling of each sequence and an ASV table was obtained based on the comparison of the Silva 16S rRNA gene database (v138). Then, species diversity analysis, principal component analysis, principal coordinate analysis, and non-metric analysis were mostly scaled through the diversity cloud analysis platform (cloud.majorbio.com (accessed on 6 December 2021)) of Shanghai Meiji Biomedical Technology Co., Ltd., Shanghai, China. The Kruskal–Wallis rank sum test was used to obtain species information with significant differences among multiple groups.

### 2.10. Detection Colonization Ability of B.longum 070103 in Mouse Feces by Quantitative Real-Time PCR

The ability of *B.longum* 070103 to colonize the mouse gut was assessed by the Light Cycler^®^ 96 real-time PCR system (Roche, Basel, Switzerland). For *B.longum* DNA amplification, the following primers were used: Forward 5′-AGCCCTGAAAGAAACAACCA-3′ and Reverse 5′-GAACAAGGCAAGGTCCAGTC-3′. DNA amplification reaction conditions were denaturation at 95 °C for 120 s, followed by 45 cycles of denaturation at 95 °C for 20 s, and annealing at 60 °C for 60 s. The data were analyzed using the built-in software. All fecal DNA of BLFM was extracted for qPCR analysis in triplicate.

### 2.11. Statistical Analysis

All data are expressed as the mean ± standard deviation with SPSS 22.0 (IBM, Armonk, NY, USA)and Graph Pad 8.2.1 software (GraphPad Software, San Diego, CA, USA) used to perform one-way analysis of variance and determine significant differences between groups; *p*-values < 0.05 were considered to be statistically significant.

## 3. Results

### 3.1. B. longum 070103 Fermented Milk Increased Glucokinase Activity

Recombinant glucokinase was obtained by purification using a Ni^2+^ affinity column (Supplementary Figure S1) and the effect of fermented milk from different bacteria on glucokinase activity was evaluated by coupling with glucose 6-phosphate dehydrogenase. *B. longum* 070103 fermented milk had a positive effect on glucokinase activation (Table 1).

### 3.2. B. longum Fermented Milk (BLFM) Improves Obesity Caused by HFHS

**Figure 1:**
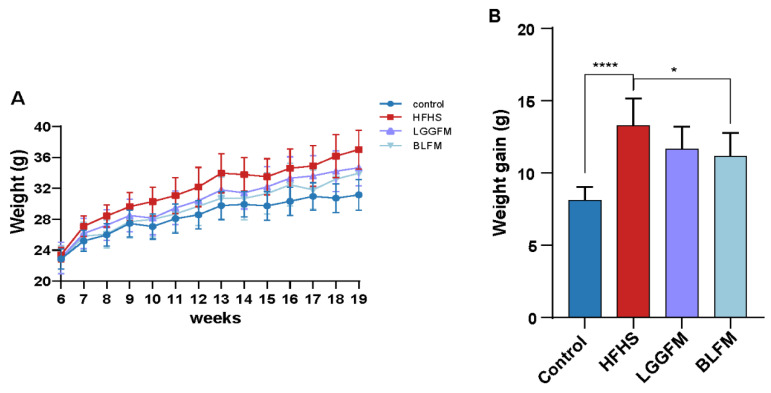
Effects of supplementing fermented milk on BW and weight gain: (**A**) BW change in mice at 13 weeks, (**B**) BW gain. Data are presented as means ± SD (n = 10) and analyzed using one-way ANOVA (* *p* < 0.05, **** *p* < 0.0001).

The BW of mice in the HFHS group significantly increased during the entire experimental period compared with the control group (Figure 1A). However, mice supplemented with LGGFM and BLFM showed a decreasing trend compared with HFHS, and BLFM significantly reduced the BW (Figure 1B). In addition, the liver, kidney, spleen, perirenal fat, and epididymal fat coefficients of the HFHS group were significantly higher than control group, but there was no significant difference in the weight of brown fat (Table 2). The liver and epididymal fat in the BLFM group were significantly reduced after intervention, while the remaining organs and tissues showed a decreasing trend compared to the HFHS group, but the difference was insignificant (Table 2).

### 3.3. BLFM Improves Hyperglycemia and Dyslipidemia in HFHS-Fed Mice

**Figure 2:**
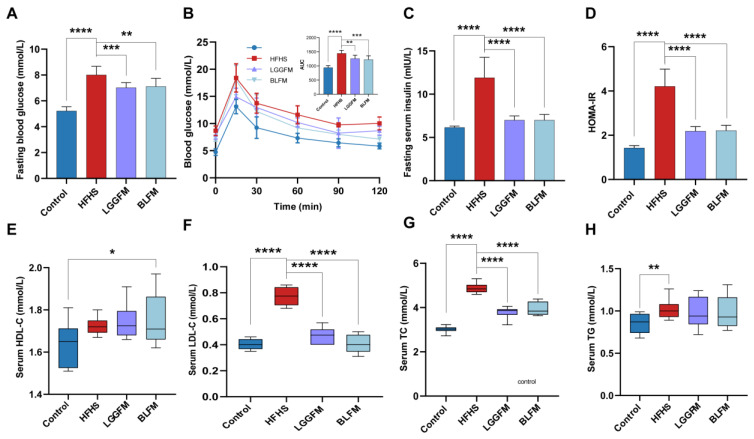
Supplementing fermented milk improves glucose and lipid metabolism disorders in mice: (**A**) fasting blood glucose, (**B**) OGTT and AUC, (**C**) fasting serum insulin, (**D**) HOMA-IR, (**E**) HDL-C, (**F**) LDL-C, (**G**) total cholesterol, (**H**) triacylglycerol. Data are presented as means ± SD (n = 10) and analyzed using one-way ANOVA (* *p* < 0.05, ** *p* < 0.01, *** *p* < 0.001, **** *p* < 0.0001).

The effect of BLFM supplementation on glucose and lipid metabolism disorders in HFHS fed mice was tested by analyzing the relevant markers. A glucose tolerance test was performed at the end of the 12th week. The fasting blood glucose, AUC (glucose tolerance), fasting insulin, HOMA-IR index, serum LDL-C, TG, and TC concentrations in the HFHS group significantly increased compared with the control group (Figure 2A–D,F,H). The HFHS group had severe glucose intolerance and dyslipidemia. In contrast, HFHS mice supplemented with LGGFM and BLFM showed significantly improved metabolic parameters after intervention compared with the HFHS group. The serum LDL-C and TC concentrations in the BLFM group significantly decreased (Figure 2F,G), the HDL-C concentration significantly increased (Figure 2E), and the TG content showed an insignificant downward trend (Figure 2H). In addition, the decreased AUC of the OGTT indicates a significant improvement in glucose tolerance. There was no significant difference between LGGFM and BLFM in improving glucose and lipid metabolism indicators, suggesting that BLFM can also improve glucose and lipid metabolism disorders compared with LGGFM.

### 3.4. BLFM Improves HFHS Induced Liver Steatosis, Inhibits White Adipose Tissue (WAT) Proliferation

**Figure 3:**
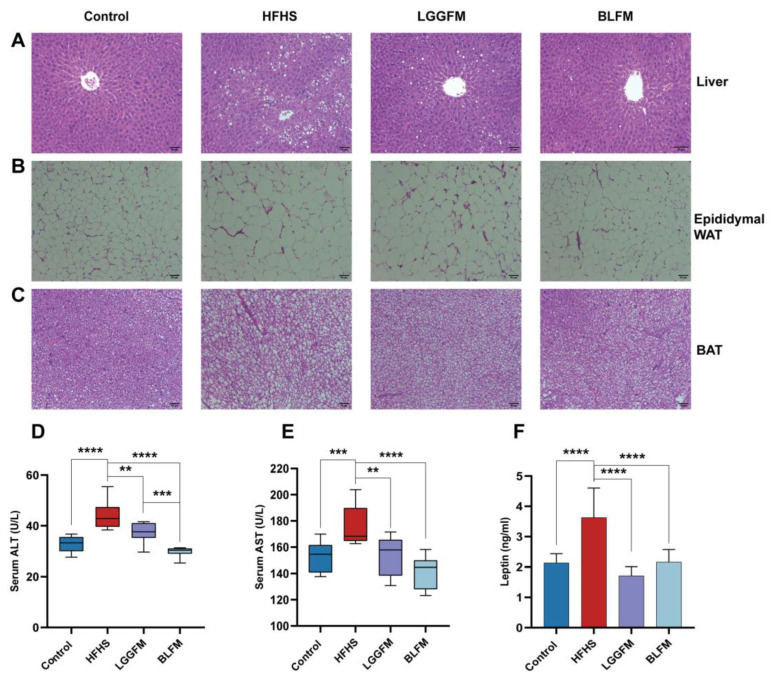
Effect of supplementing fermented milk on pathological changes of liver, adipose tissue and colon tissue in mice: (**A**–**C**) H&E of liver tissue sections, epididymal white adipose tissue (epididymal WAT), brown fat (BAT), (**D**,**E**) glutamic pyruvic transaminase (ALT) and glutamic oxalacetic transaminase (AST), (**F**) leptin. Data are presented as means ± SD (n = 10) and analyzed using one-way ANOVA (** *p* < 0.01, *** *p* < 0.001, **** *p* < 0.0001).

A HFHS diet caused liver steatosis of the HFHS group through obvious lipid accumulation according to histological analysis of H&E-stained liver (Figure 3A), while intervention with LGGFM and BLFM significantly reduced the size and number of lipid droplets. ALT and AST are biomarkers for hepatotoxicity and early metabolic syndrome [32,33,34]. Serum ALT and AST levels were significantly higher in the HFHS group than in the control group. Serum ALT and AST levels were significantly reduced in mice supplemented with LGGFM and BLFM, and BLFM was reduced to a greater degree than LGGFM (Figure 3D,E). This shows that BLFM supplementation reduces liver damage caused by HFHS. Histopathological analysis revealed that the lipid droplet size in the epididymal fat and brown adipose tissue of the HFHS group was significantly larger than that of the control group. The adipose tissue had smaller lipid droplets after intervention of the HFHS group with LGGFM and BLFM treatment (Figure 3B,C). High plasma leptin levels are correlated with body fat mass [35], with leptin resistance caused by decreased tissue sensitivity to prolonged high circulating leptin levels [36] and increased susceptibility to obesity. Supplementation with LGGFM and BLFM significantly reduced leptin resistance caused by obesity in mice fed a HFHS diet (Figure 3F).

### 3.5. BLFM Modulates Gut Microbiota

**Figure 4:**
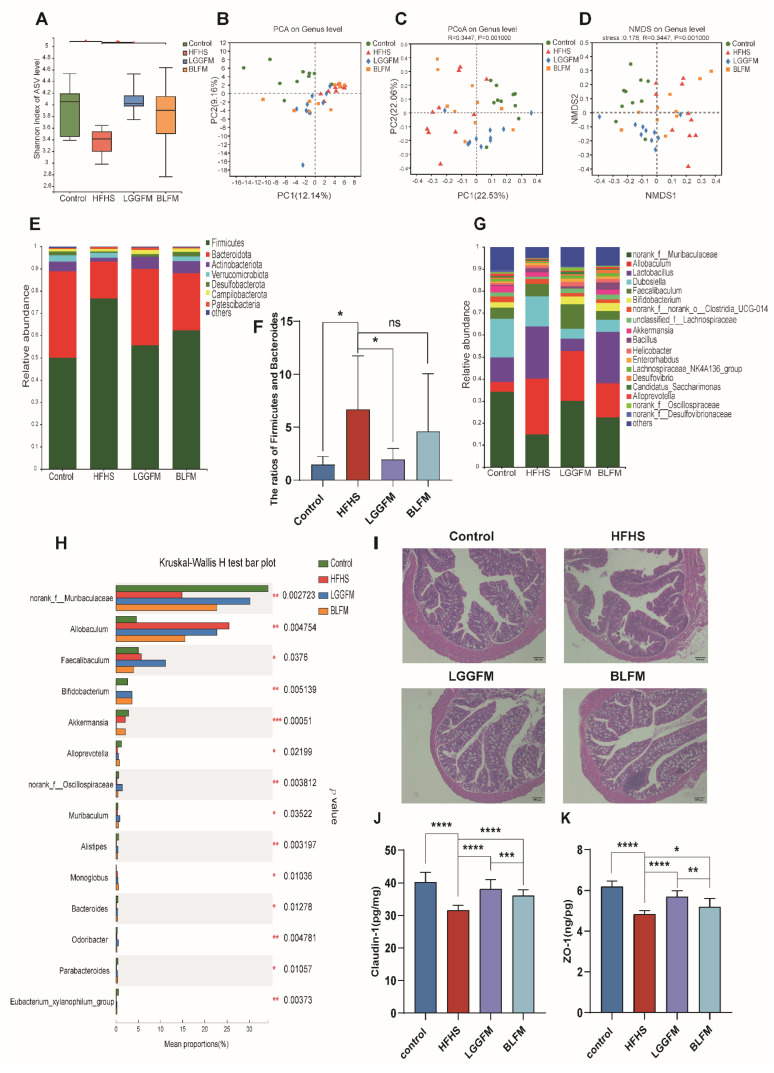
FM modulates the gut microbiota: (**A**) Shannon index, (**B**–**D**) Plots of PCoA, NMDS, and PCA scores based on the Bray-Curties distance algorithm, (**E**) Relative abundance of gut microbiota at the phylum level, (**F**) Ratio of Firmicutes to Bacteroidetes, (**G**) Relative abundance of gut microbiota at the genus level, (**H**) Relative abundance of bacterial communities at the genus level among different groups, (**I**) H&E of colon tissue sections, (**J**) The content of Claudin-1 in the colon, (**K**) The content of ZO-1 in the colon. Data are presented as means ± SD (n = 10) and analyzed using one-way ANOVA (ns, not significant, * *p* < 0.05, ** *p* < 0.01, *** *p* < 0.001, **** *p* < 0.0001).

Since glucose and lipid metabolism disorder is closely related to gut microbiota, this study examined the relationship between the supplementation of fermented milk in the diet and the gut microbiota. The significantly lower Shannon index in the HFHS group compared with that in the control group indicates that the community diversity and richness of the samples were lower in the HFHS group (Figure 4A). A significant increase in the gut microbiota diversity was observed after LGGFM and BLFM intervention (Figure 4B–D) with Firmicutes, *Bacteroides*, and Actinomycetes being the most abundant taxa. The F/B ratio in the HFHS group was significantly increased compared with the control group. LGGFM and BLFM intervention significantly and insignificantly decreased the F/B, respectively (Figure 4E,F). The main microbiota consisted of *norank_f_Muribaculaceae, Allobaculum*, *Lactobacillus*, *Dubosiella*, *Faecalibaculum*, and *Bifidobacterium* (Figure 4G). The abundance of *norank_f_Muribaculaceae*, *Bifidobacterium*, *Alloprevotella*, *norank_f_Oscillospiraceae*, *Alistipes*, *Bacteroides*, and *Parabacteroides* in the HFHS group was lower than those in the control group, while *Allobaculum* and *Faecalibaculum* abundance was higher than those of the control group. Supplementation of LGGFM and BLFM to the HFHS group significantly increased the abundance of *norank_f_Muribaculaceae*, *Bifidobacterium*, and other bacteria (Figure 4H). At the same time, the feces of mice fed with BLFM were evaluated to detect the colonization ability of *B. longum*, and the number was 4.36 ± 0.013 lg/g of feces (Supplementary Figure S2).

In addition, the HFHS group had sparse intestinal villi and the glands were reduced compared with the control group indicating that HFHS caused colon damage. LGGFM and BLFM intervention reverted the intestinal villi to their dense form and significantly the gland was restored to a similar size as that of the control (Figure 4I). ELISA showed that colonic ZO-1 and Claudin-1 levels were significantly lower in the HFHS group than those in the control group, indicating that the integrity of the intestinal mucosal barrier was destroyed with a HFHS diet. ZO-1 and Claudin-1 significantly increased after LGGFM and BLFM intervention compared with the HFHS group (Figure 4J,K). Therefore, BLFM intervention significantly improved mucosal permeability and restored the integrity of the intestinal mucosal barrier by regulating tight junction proteins.

### 3.6. BLFM Improves HFHS Induced Fecal Metabolites in Mice

**Figure 5:**
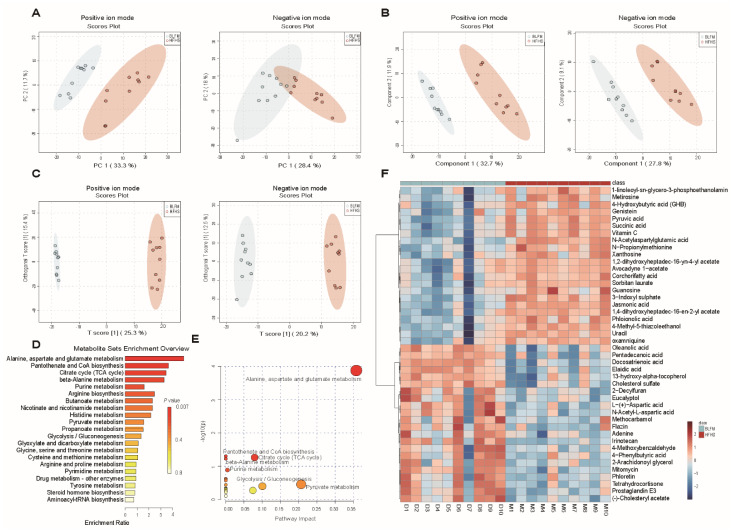
Supplementation of fermented milk changes the fecal metabolites and related KEGG pathways of mice on HFHS diet: (**A**) principal component analysis (PCA), (**B**) Partial Least Squares Discriminant Analysis (PLS-DA), (**C**) Orthogonal Partial Least Squares Discriminant Analysis (OPLS-DA), (**D**) KEGG pathway enrichment histogram, (**E**) Overview of pathway analysis, (**F**) heatmap of differential metabolites.

Changes in the gut microbiota inevitably affect the biotransformation of dietary components by the gut microbiota. The contents of metabolites in feces in a HFHS diet following intervention with BLFM were analyzed using a non-targeted metabolomics approach with LC-MS. The analysis of PCA (Figure 5A), PLS-DA (Figure 5B), and OPLS-DA (Figure 5C) showed that the distribution of the samples was significantly different, suggesting that the HFHS diet significantly changed the fecal metabolites of mice compared with the normal diet. A VIP value > 1.5, FC > 2, and *t* test *p* value < 0.01 revealed 44 different metabolites between the HFHS group and BLFM group according to the discriminant analysis of the orthogonal partial least squares method (Supplementary Table S2). Enrichment analysis and pathway enrichment analysis of the 44 different metabolites showed that most of the metabolites were concentrated in the metabolism of alanine, aspartic acid, glutamic acid, pantothenic acid, coenzyme A biosynthesis, citric acid cycle, β-alanine metabolism, purine metabolism, arginine biosynthesis, and butyrate metabolism (Figure 5D). The contribution ratios of different metabolites to metabolic pathways are shown in Figure 5E. After intervention with BLFM, the abundance of 22 feces metabolites significantly increased, including 4-phenylbutyric acid, oleanolic acid, pentadecanoic acid, docosatrienoic acid, 13-hydroxy-α-tocopherol, and L-(+)-aspartic acid. Meanwhile, a significant reduction in the abundance of 22 metabolites was observed, including ɣ-hydroxybutyric acid (GHB), 3-indoxyl sulphate, and other substances. The heat map analysis illustrates the altered levels of the differential metabolites in the HFHS and BLFM groups (Figure 5F).

### 3.7. Correlation among Gut Microbiota, Fecal Metabolites, and Pathological Indicators

**Figure 6:**
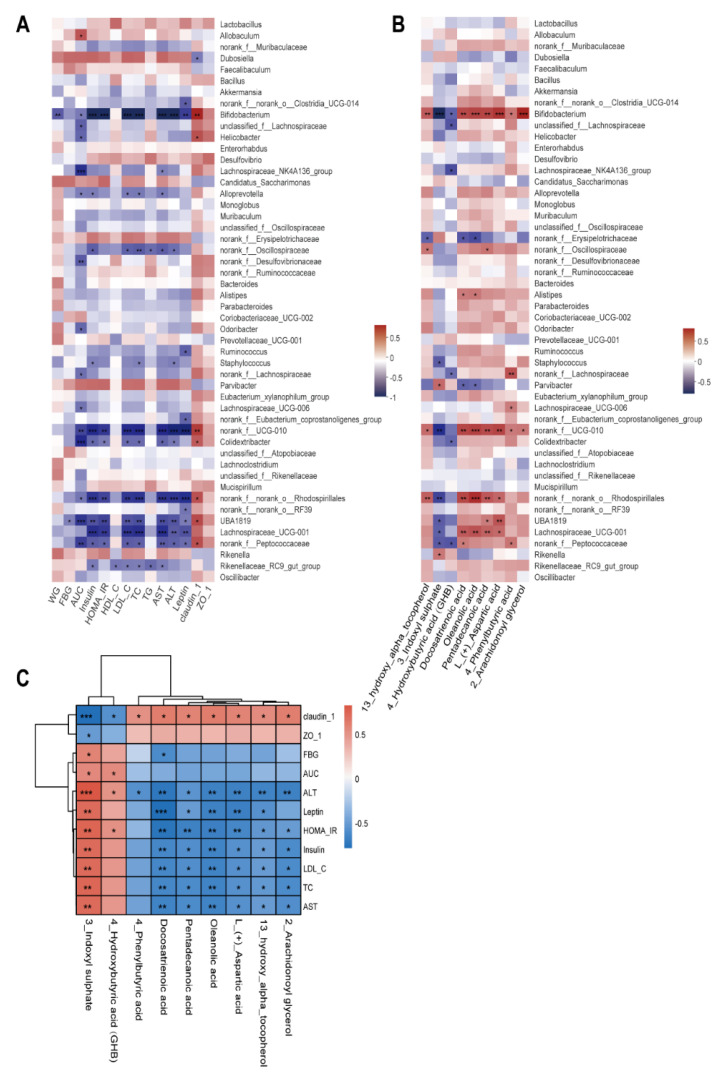
Correlation analysis of gut microbiota, fecal metabolites, and glucose and lipid metabolism indexes: (**A**) Heatmap of correlations between metabolic parameters and the top 50 genera in gut microbiota abundance, (**B**) Heatmap of correlations between nine important differential fecal metabolites and the top 50 genera in gut microbiota abundance, (**C**) Heatmap of correlations between metabolic parameters and important differential fecal metabolites. The legend on the right is the color range for different R values, red for positive correlation and blue for negative correlation. (* *p* < 0.05, ** *p* < 0.01, *** *p* < 0.001).

Spearman’s correlation coefficient analysis revealed significant correlation between 14 physiological and biochemical indicators and the top 50 genera of gut microbiota in the HFHS and BLFM groups. In particular, *Bifidobacterium* was negatively correlated with WG, AUC, insulin, HOMA-IR, HDL-C, LDL-C, TC, TG, AST, ALT, and leptin, and positively correlated with claudin-1 (Figure 6A). The abundances of *norank_f__norank_o__Rhodospirillales*, *norank_f__Peptococcaceae*, and *norank_f__UCG-010* were negatively correlated with AUC, insulin, HOMA-IR, LDL-C, TC, AST, ALT, and leptin, and positively correlated with claudin-1. The abundances of *UBA1819*, *Lachnospiraceae_UCG-001*, and *Colidextribacter* were negatively correlated with insulin, HOMA-IR, LDL-C, TC, AST, and ALT. Claudin-1 was positively correlated with the abundances of *UBA1819* and *Colidextribacter*, while AUC was negatively correlated with these bacteria. Leptin was negatively correlated with *UBA1819* and *Lachnospiraceae_UCG-001*.

Correlation analysis of the abundance of fecal metabolites and gut microbiota showed that *Bifidobacterium* was positively correlated with 13-hydroxy-alpha-tocopherol, docosatrienoic acid, oleanolic acid, pentadecanoic acid, L-(+)-aspartic acid, 4-phenylbutyric acid, and 2-arachidonoylglycerol, while a negative correlation was observed with *Bifidobacterium* and 3-indoxyl sulphate or GHB. The abundance of *norank_f__UCG-010* and *norank_f__norank_o__Rhodospirillales* was positively correlated with 13-hydroxy-alpha-tocopherol, docosatrienoic acid, oleanolic acid, pentadecanoic acid, and L-(+)-aspartic acid, while a negatively correlation was observed with 3-indoxyl sulphate. It is worth noting that the abundances of *Rikenella* and *Parvibacter* were proportional to 3-indoxyl sulphate (Figure 6B).

The nine metabolites were significantly correlated with at least one physiological and biochemical index with a total of 65 significantly correlations. Among them, 3-indoxyl sulphate was positively correlated with FBG, AUC, insulin, HOMA-IR, LDL-C, TC, AST, ALT, and leptin. There was a negative correlation between the expression of claudin-1 and ZO-1. Claudin-1 and ALT were negatively correlated with 13-hydroxy-alpha-tocopherol, docosatrienoic acid, oleanolic acid, pentadecanoic acid, L-(+)-aspartic acid, 4-phenylbutyric acid, and 2-arachidonoylglycerol. Meanwhile, they were positively correlated with 3-indoxyl sulphate and GHB (Figure 6C). These results indicate that BLFM reduces the production of 3-indoxyl sulphate by increasing the abundance of bifidobacteria and metabolites, such as docosatrienoic acid and oleanolic acid. This partially regulates the gut microbiota, improves the intestinal barrier, and alleviates glucose and lipid metabolism disorders caused by HFHS.

## 4. Discussion and Conclusions

The health benefits of fermented dairy products are closely related to the strains and metabolites produced during fermentation, especially the presence of active ingredients such as probiotics, peptides, conjugated linoleic acid, etc. [37]. Glucokinase is the rate-limiting enzyme in the first step of glycolysis. A small change in its activity alters the glucose-stimulated insulin secretion threshold of pancreatic β-cells and regulates the steady state of glucose [38]. Supplementation of probiotic fermented milk has a positive effect on obesity and diabetes [39,40,41,42].

In this study, a screening system was constructed by the heterologous expression and purification of human liver glucokinase in *E. coli*. Secondly, probiotic strains isolated from the feces of healthy individuals were used to ferment milk. Finally, the supernatant of fermented milk was used to screen the fermented milk with glucokinase activation. This research is the first attempt to screen fermented milk with probiotics that activate glucokinase. BLFM significantly improved glucose and lipid metabolism disorders in mice caused by high fat and high sugar levels by regulating gut microbiota and changing metabolite composition.

*B. longum* 070103 fermented milk significantly reduced the weight gain induced by high-fat and high-sugar diets, improved the steady state of glucose and lipid metabolism, and reduced fatty liver degeneration and white fat proliferation. This is consistent with other studies on fermented milk with probiotics [22,43,44,45]. This study also proved that BLFM significantly increased the production of claudin-1 and ZO-1 and repaired the intestinal barrier. Impaired intestinal barrier integrity is considered to be an important cause of various systemic metabolic diseases such as insulin resistance, obesity, and type 2 diabetes [46,47,48,49]. The improvement of the intestinal barrier was consistent with the improvement in the colon tissue observed by H & E staining. The intestinal barrier is closely related to the gut microbiota and intestinal metabolites. Therefore, it is possible that the regulation of intestinal microbes leads to changes in metabolites, repairs the intestinal barrier, and improves the disorder of glucose and lipid metabolism [50]. In our mouse model, the overall structure of the gut microbiota changes after BLFM intervention, thereby improving the imbalance of the microbiota induced by HFHS diet. Moreover, changes in the gut microbiota structure are related to physiological and biochemical indicators of glucose and lipid metabolism disorders.

Differences in diet can lead to changes in the gut microbiota. Gut microbiota imbalance is the most important factor affecting the intestinal barrier and is associated with various metabolic diseases [51]. The intake of different food components has a significant effect on microbe levels [2]. For example, the intake of navy beans and broccoli reduced the F/B ratio [9,52], and our supplementation with BLFM similarly reduced the F/B ratio compared. Therefore, we can intervene in the treatment of certain diseases through food. The high-fat/carbohydrate diet significantly reduces the abundance and diversity of the gut microbiota with significantly reduced *Bacteroides* leading to obesity and dyslipidemia [53]. In contrast, increasing the abundance of such bacteria alleviates disease. Supplementation of obese women with inulin-type fructans reduces the metabolic risk factors associated with higher fecal short-chain fatty acid concentrations, and significantly enriches *B. longum*, pseudo-chain *Bifidobacterium*, *Bifidobacterium adolescentis*, and *Bacillus*, with *B. longum* negatively correlated with serum lipopolysaccharide and endotoxin [54]. *Bifidobacterium* is considered a target for regulating obesity microbiota [55]. In this study, Spearman’s correlation analysis was used to determine the degree of correlation between changes in gut microbiota following supplementation with fermented milk, and host metabolic parameters. Supplementation with fermented milk significantly increased the abundance of *norank_f_Muribaculaceae*, *Bifidobacterium*, *Alloprevotella*, *norank_f__Oscillospiraceae*, *Alistipes*, *Bacteroides*, and *Parabacteroides*. These bacteria alleviate glucose and lipid metabolism disorders caused by obesity and a high-fat diet [56,57,58,59,60] by the production of succinic acid and secondary bile acids. In contrast, *Allobaculum* and *Faecalibaculum* were significantly enriched in the HFHS group, which are associated with the development of metabolic diseases [3,61], and supplementation with fermented milk reduced the abundance of these bacteria. In this study, supplementation of fermented milk to the mouse diet significantly increased the abundance of *Bifidobacteria* which alleviating metabolic disorders. *Bifidobacteria* were negatively correlated with WG, AUC, insulin, HOMA-IR, HDL-C, LDL-C, TC, TG, AST, ALT, and leptin and positively correlated with claudin-1. In summary, supplementation with BLFM specifically regulates key gut microbiota, thereby improving host metabolism.

There is a high correlation between the gut microbiome and the fecal metabolome which is associated with host health and diet [62]. A high-fat diet destroys the intestinal barrier through various mechanisms such as the destruction of the tight junctions of the intestinal epithelium, imbalance of gut microbiota, oxidative stress, and inflammation [63]. The permeability of the intestinal barrier aggravates metabolic disorders caused by a high-fat diet [64,65]. Hyperglycemia increases intestinal permeability in obese and diabetic mice leading to microbial metabolites entering the host and increasing the risk of infection [66]. In this study, the high-fat diet group significantly increased the content of 3-indoxyl sulphate and 4-hydroxybutyric acid in the feces. Moreover, 3-indoxyl sulphate was strongly correlated with all metabolic parameters. This metabolite inhibits DPR1 (dynamin-related protein 1) in intestinal epithelial cells by enhancing IRF1 (interferon regulatory factor 1) expression, inhibiting mitochondrial autophagy, and inducing intestinal barrier damage [67]. This substance is also considered biomarkers for patients with type 2 diabetic atherosclerosis [68]. GHB is an inhibitory neurotransmitter that can inhibit glucagon production. However, studies have shown that it can stimulate the secretion of human glucagon in contrast to impaired regulation of glucagon secretion in type 2 diabetes patients with hyperglucagonemia [69]. BLFM supplementation significantly reduced 3-indoxyl sulphate and 4-hydroxybutyric acid levels while increasing oleanolic acid, pentadecanoic acid, and L- (+)-aspartic acid. These metabolites are related to the alleviation of glucose and lipid metabolism disorders [70,71,72,73].

In summary, BLFM counteracted glucose and lipid metabolism disorders caused by high fat and sugar by adjusting the gut microbiota, changing fecal metabolites, and repairing the intestinal barrier. BLFM regulated the gut microbiota, increased F/B, significantly increased the abundance of *Bifidobacteria*, and promoted the production of pentadecanoic acid, 4-phenylbutyric acid, and docosatrienoic acid in mice with metabolic disorders induced by diet. Trienoic acid, oleanolic acid, 13-hydroxy-α-tocopherol, and L-(+)-aspartic acid production significantly reduced GHB and 3-indoxyl sulphate and repaired the damaged intestinal barrier. These results indicate that *B. longum* 070103 fermented milk can be considered a dietary supplement to alleviate glucose and lipid metabolism disorders.

## Figures and Tables

**Table 1:** Screening of glucokinase activator in vitro.

Strain Name	Strain Number	Strain Source	Activation Multiple
*Bifidobacterium longum*	070103	human feces	1.16 ± 0.03
*Bifidobacterium bifidum*	070507	human feces	1.10 ± 0.05
*Bifidobacterium longum*	070106	human feces	0.81 ± 0.06
*Lactobacillus delbrueckii*	R37	fermented dairy products	1.09 ± 0.08
*Lactiplantibacillus* *plantarum*	R51	fermented dairy products	1.04 ± 0.08
*Lactiplantibacillus* *plantarum*	R95	fermented dairy products	1.07 ± 0.04
*Lactiplantibacillus* *plantarum*	1–134	fermented dairy products	1.06 ± 0.03
*Levilactobacillus* *brevis*	SR52-2	salted fish	1.05 ± 0.03
*Lactiplantibacillus* *plantarum*	R22	yogurt	0.89 ± 0.06
*Lactiplantibacillus* *plantarum*	0101127-4	pickle	0.93 ± 0.07

The information on the remaining 77 strains of lactic acid bacteria is placed in Supplementary Table S1.

**Table 2:** Indexes of organ and fat coefficient of mice in each group (%).

Group	Control	HFHS	LGGFM	BLFM
Liver index	3.90 ± 0.05 ^a^	4.20 ± 0.13 ^b^	3.78 ± 0.06 ^a^	3.86 ± 0.08 ^a^
Renal index	1.26 ± 0.03 ^a^	1.40 ± 0.03 ^b^	1.22 ± 0.03 ^a^	1.36 ± 0.02 ^b^
Spleen index	0.27 ± 0.01 ^a^	0.30 ± 0.01 ^b^	0.26 ± 0.01 ^a^	0.28 ± 0.01 ^ab^
Perirenal fat coefficient	0.44 ± 0.06 ^a^	1.35 ± 0.18 ^bc^	1.53 ± 0.21 ^c^	1.01 ± 0.09 ^b^
Epididymal fat coefficient	1.45 ± 0.16 ^a^	4.31 ± 0.35 ^c^	4.10 ± 0.43 ^c^	3.14 ± 0.30 ^b^
Brown fat coefficient	0.39 ± 0.03 ^a^	0.43 ± 0.03 ^a^	0.36 ± 0.02 ^a^	0.36 ± 0.04 ^a^

Values are means ± SD, n = 10. Means in a row without a common letter differ. Values with superscript letters ^a^, ^b^ and ^c^ are significantly different across rows (*p* < 0.05).

## Data Availability

16S amplicon data from animal feces of C57/BL6J mice https://submit.ncbi.nlm.nih.gov/subs/sra/SUB12024050/overview (accessed on 7 September 2022) Submission ID: SUB12024050, BioProject ID: PRJNA877441.

## Supplementary Materials

The following supporting information can be downloaded at: https://www.mdpi.com/article/10.3390/nu14194050/s1, Figure S1: SDS-PAGE of G6PDH and GK. Figure S2: Colonization ability of *Bifidobacterium longum* 070103. Table S1: Screening of glucokinase activator in vitro (77 samples). Table S2: Differential metabolites between BLFM and HFHS groups.
