# Supplementary material for: Bifidobacterium longum 070103 Fermented Milk Improve Glucose and Lipid Metabolism Disorders by Regulating Gut Microbiota in Mice
Source: Nutrients. 2022 Sep 29;14(19):4050. doi: 10.3390/nu14194050 (PMC9573661; doi:10.3390/nu14194050)
Supplement: Supplementary file 1 [file nutrients-14-04050-s001.zip › nutrients-1931714-supplementary.pdf]

Supplementary Materials

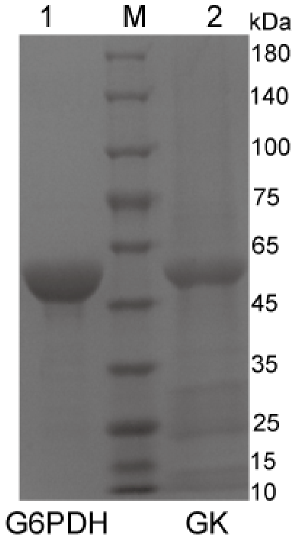

Figure S1. SDS-PAGE of G6PDH and GK. Lanes 1: G6PDH. Lanes 2: GK. Lane M: Protein Marker.

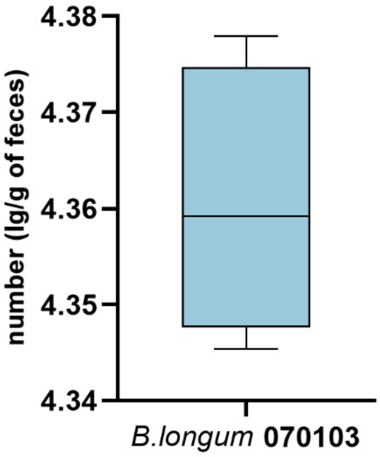

Figure S2. Colonization ability of *Bifidobacterium longum* 070103.

Table S1. Screening of glucokinase activator in vitro (77 samples).

Strain name	Strain number	Strain source	Activation multiple
Bifidobacterium longum	070103	human feces	1.16±0.03
Bifidobacterium longum	050104	human feces	0.96±0.03
Bifidobacterium longum	050402	human feces	1.14±0.05
Bifidobacterium longum	050101	human feces	0.88±0.13
Bifidobacterium longum	050503	human feces	1.10±0.07
Bifidobacterium longum	050504	human feces	0.88±0.03
Bifidobacterium longum	050505	human feces	0.88±0.01
Bifidobacterium longum	050506	human feces	0.88±0.01
Bifidobacterium longum	050507	human feces	1.01±0.07
Bifidobacterium bifidum	070202	human feces	0.75±0.08
Bifidobacterium bifidum	070304	human feces	0.86±0.04
Bifidobacterium bifidum	070305	human feces	0.81±0.02
Bifidobacterium bifidum	070310	human feces	0.77±0.05
Bifidobacterium longum	070412	human feces	0.83±0.02
Bifidobacterium bifidum	070413	human feces	0.83±0.08
Bifidobacterium bifidum	070501	human feces	1.03±0.05
Bifidobacterium bifidum	070503	human feces	0.9±0.02
Bifidobacterium bifidum	070504	human feces	0.83±0.08
Bifidobacterium bifidum	070506	human feces	0.96±0.08
Bifidobacterium bifidum	070510	human feces	0.87±0.08
Bifidobacterium bifidum	070512	human feces	0.93±0.03
Bifidobacterium bifidum	070516	human feces	1.04±0.02
Lactiplantibacillus plantarum	1-23	fermented dairy products	0.97±0.03
Lactiplantibacillus plantarum	1-25	fermented dairy products	1.07±0.03
Lactiplantibacillus plantarum	1-28	fermented dairy products	1.01±0.03
Limosilactobacillus fermentum	3030-1	human feces	1.00±0.08

Lactiplantibacillus plantarum	30-4	olivetta gp leaves	0.87±0.11
Lactiplantibacillus plantarum	B82-1	human feces	0.95±0.08
Lactiplantibacillus plantarum	D95	human feces	1.05±0.03
Lactiplantibacillus plantarum	E4	human feces	0.96±0.04
Lactiplantibacillus plantarum	E53	human feces	0.94±0.06
Lactiplantibacillus plantarum	E57	human feces	0.97±0.03
Lactiplantibacillus plantarum	SR12-1	salted fish	0.94±0.06
Lactiplantibacillus plantarum	E77	human feces	0.83±0.06
Lactiplantibacillus plantarum	E82	human feces	0.96±0.06
Lactiplantibacillus plantarum	E88-1	human feces	1.04±0.19
Lactiplantibacillus plantarum	H6	human feces	0.73±0.02
Lactiplantibacillus pentosus	SR37-3	salted fish	0.93±0.01
Lactiplantibacillus plantarum	H88	human feces	1.05±0.01
Lactiplantibacillus plantarum	J14	human feces	1.01±0.02
Limosilactobacillus fermentum	J21	human feces	0.94±0.06
Lactiplantibacillus pentosus	J42	human feces	1.04±0.08
Lactiplantibacillus plantarum	K69	human feces	0.7±0.01
Limosilactobacillus reuteri	Q10	fermented dairy products	0.35±0.04
Lactiplantibacillus plantarum	L2	human feces	0.97±0.05
Lactiplantibacillus plantarum	L43	human feces	0.9±0.02
Limosilactobacillus fermentum	L61	human feces	0.92±0.08
Lactiplantibacillus plantarum	L81	human feces	0.74±0.05
Limosilactobacillus fermentum	Q40	yogurt	1.02±0.03
Lactiplantibacillus plantarum	L85	human feces	0.78±0.04
Limosilactobacillus fermentum	P15	yogurt	1.01±0.06
Limosilactobacillus fermentum	L86	human feces	0.96±0.08
Lactiplantibacillus plantarum	L86-1	human feces	0.93±0.01
Lactiplantibacillus plantarum	L3	human feces	0.95±0.03
Lactiplantibacillus plantarum	M2	human feces	1.08±0.04

Lactiplantibacillus plantarum	M39	human feces	1.02±0.05
Lactiplantibacillus plantarum	M4	human feces	0.83±0.06
Lactiplantibacillus pentosus	M40	human feces	0.99±0.03
Lactiplantibacillus plantarum	M41-2	human feces	0.93±0.02
Limosilactobacillus fermentum	P30	fermented dairy products	0.8±0.06
Lactiplantibacillus plantarum	Q17	fermented dairy products	0.62±0.44
Limosilactobacillus oris	Q5	fermented dairy products	0.81±0.03
Lactiplantibacillus plantarum	Q63	fermented dairy products	0.81±0.03
Lactiplantibacillus plantarum	R25	yogurt	1.02±0.01
Limosilactobacillus fermentum	R33	fermented dairy products	0.89±0.06
Limosilactobacillus fermentum	R38	fermented dairy products	1.05±0.08
Lactobacillus delbrueckii	R49	fermented dairy products	1.02±0.05
Limosilactobacillus fermentum	R52	fermented dairy products	1.03±0.04
Lactiplantibacillus plantarum	R53	fermented dairy products	0.96±0.03
Limosilactobacillus fermentum	R54	fermented dairy products	0.97±0.05
Lactiplantibacillus plantarum	R55	fermented dairy products	0.98±0.04
Lactiplantibacillus plantarum	R57	fermented dairy products	1.07±0.04
Lactiplantibacillus plantarum	R59	fermented dairy products	0.04±0.02
Lacticaseibacillus casei	010203-5	fermented dairy products	0.08±0.03
Lacticaseibacillus rhamnosus	R60	yogurt	1.04±0.04
Lacticaseibacillus rhamnosus	R62	yogurt	0.93±0.01
Lactiplantibacillus plantarum	R64	yogurt	0.89±0.05
Lactiplantibacillus plantarum	0101184-1	fermented fruit and vegetable products	1.02±0.03

Table S2. Differential metabolites between BLFM and HFHS groups.

Comparsion	NO.	ESI Mode	putative Compound	Main Class	Formula	Molecular Weight	RT (min)	VIP	*p*-Value	log2(FC)	Modulation
BLFMvsHFHS	1	−	Adenine	Imidazopyrimidines	$C_5H_5N_5$	135.05	2.79	1.61	0.00031735	1.6807	up
	2	−	Vitamin C	Dihydrofurans	$C_6H_8O_6$	176.03	0.89	1.95	1.31E-06	-2.2664	down
	3	−	Guanosine	Purine nucleosides	$C_{10}H_{13}N_5O_5$	283.09	2.84	1.52	0.0012305	-1.5726	down
	4	+	L-(+)-Aspartic acid	Carboxylic acids and derivatives	$C_4H_7NO_4$	133.04	1.56	1.58	9.75E-08	2.2435	up
	5	−	Pyruvic acid	Keto acids and derivatives	$C_3H_4O_3$	88.02	0.84	1.94	9.47E-07	-2.4024	down
	6	−	Succinic acid	Carboxylic acids and derivatives	$C_4H_6O_4$	118.03	0.83	1.95	1.21E-06	-3.3426	down
	7	−	Xanthosine	Purine nucleosides	$C_{10}H_{12}N_4O_6$	284.08	3.05	1.55	0.0011398	-2.703	down
	8	−	Uracil	Diazines	$C_4H_4N_2O_2$	112.03	1.28	1.64	0.00033587	-1.177	down
	9	+	4-Phenylbutyric acid	Benzene and substituted derivatives	$C_{10}H_{12}O_2$	164.08	22.00	1.55	0.00010391	1.2452	up
	10	−	Elaidic acid	Fatty Acyls	$C_{18}H_{34}O_2$	282.26	22.91	1.60	0.00056094	2.4021	up

11	−	Cholesterol sulfate	Steroids and steroid derivatives	$C_{27}H_{46}O_4S$	466.31	19.97	1.64	0.00042296	2.7085	up
12	−	3-Indoxyl sulphate	Organic sulfuric acids and derivatives	$C_8H_7NO_4S$	213.01	1.67	2.01	1.51E-07	-3.34	down
13	−	4-Hydroxybutyric acid (GHB)	Fatty Acyls	$C_4H_8O_3$	104.05	0.94	1.67	0.00032566	-1.3995	down
14	+	N-Acetyl-L-aspartic acid	Carboxylic acids and derivatives	$C_6H_9NO_5$	175.05	1.56	1.62	2.38E-07	2.1142	up
15	−	Pentadecanoic acid	Fatty Acyls	$C_{15}H_{30}O_2$	242.22	20.30	1.53	0.00081062	1.7875	up
16	+	Tetrahydrocortisone	Steroids and steroid derivatives	$C_{21}H_{32}O_5$	346.21	7.51	1.61	5.86E-07	2.0958	up
17	−	N-Acetylaspartylglutamic acid	Carboxylic acids and derivatives	$C_{11}H_{16}N_2O_8$	304.09	3.00	1.82	1.52E-05	-3.3904	down
18	−	Oleanolic acid	Prenol lipids	$C_{30}H_{48}O_3$	456.36	22.95	1.55	0.00059909	2.3095	up
19	−	Prostaglandin E3	Fatty Acyls	$C_{20}H_{30}O_5$	350.21	4.26	1.75	2.10E-05	2.0429	up
20	−	Docosatrienoic acid	Fatty Acyls	$C_{22}H_{38}O_2$	334.29	23.80	1.69	0.00012264	3.2507	up
21	−	Genistein	Isoflavonoids	$C_{15}H_{10}O_5$	270.05	8.26	1.71	0.00013244	-1.5787	down

22	−	Phloretin	Linear 1,3-diarylpropanoids	$C_{15}H_{14}O_5$	274.09	5.28	1.52	0.00057359	1.3074	up
23	+	(-)-Cholesteryl acetate	Steroids and steroid derivatives	$C_{29}H_{48}O_2$	428.37	17.42	1.81	5.72E-05	2.9644	up
24	+	Eucalyptol	Oxanes	$C_{10}H_{18}O$	136.13	16.69	1.51	5.57E-05	2.0427	up
25	+	2-Arachidonoyl glycerol	Endocannabinoids	$C_{23}H_{38}O_4$	378.28	19.82	1.75	9.81E-05	1.9384	up
26	−	1-linoleoyl-sn-glycero-3-phosphoethanolamine	Glycerophospholipids	$C_{23}H_{44}NO_7P$	477.29	10.32	1.54	0.0010681	-2.5092	down
27	−	13-hydroxy-alpha-tocopherol	Prenol lipids	$C_{29}H_{50}O_3$	446.38	24.36	1.53	0.0011974	2.0121	up
28	+	Mitomycin	Indoles and derivatives	$C_{15}H_{18}N_4O_5$	334.13	0.96	1.75	1.22E-09	2.025	up
29	+	Methocarbamol	Phenol ethers	$C_{11}H_{15}NO_5$	241.09	1.13	1.58	1.53E-08	1.2816	up
30	+	Irinotecan	Camptothecins	$C_{33}H_{38}N_4O_6$	586.28	8.38	1.51	1.61E-05	3.5958	up
31	−	Metirosine	Phenylpropanoic acids	$C_{10}H_{13}NO_3$	195.09	4.77	1.68	0.0001685	-2.75	down
32	+	oxamniquine	Quinolines and derivatives	$C_{14}H_{21}N_3O_3$	279.16	5.95	1.82	3.10E-07	-1.2398	down

33	+	4-Methoxybenzaldehyde	Benzene and substituted derivatives	$C_8H_8O_2$	136.05	13.23	1.74	2.58E-05	1.6731	up
34	−	Sorbitan laurate	Fatty Acyls	$C_{18}H_{34}O_6$	346.24	5.14	1.76	8.04E-05	-1.6856	down
35	+	1,4-dihydroxyheptadec-16-en-2-yl acetate	Fatty Acyls	$C_{19}H_{36}O_4$	310.25	14.56	1.57	3.90E-05	-2.7557	down
36	+	Avocadyne 1-acetate	Fatty Acyls	$C_{19}H_{34}O_4$	308.23	18.69	1.52	8.67E-05	-1.8866	down
37	+	1,2-dihydroxyheptadec-16-yn-4-yl acetate	Fatty Acyls	$C_{19}H_{34}O_4$	326.25	18.69	1.55	6.34E-05	-1.8123	down
38	+	2-Decylfuran	Heteroaromatic compounds	$C_{14}H_{24}O$	208.18	10.11	1.76	2.01E-05	2.7732	up
39	+	Jasmonic acid	Fatty Acyls	$C_{12}H_{18}O_3$	210.13	5.71	1.54	3.07E-07	-2.3369	down
40	+	4-Methyl-5-thiazoleethanol	Azoles	C_6H_9NOS	143.04	2.95	1.87	2.92E-07	-2.6102	down
41	+	Flazin	Harmala alkaloids	$C_{17}H_{12}N_2O_4$	308.08	7.77	1.75	1.03E-05	2.1127	up
42	−	Phloionolic acid	Fatty Acyls	$C_{18}H_{36}O_5$	332.26	6.92	1.68	0.00019785	-1.9554	down
43	−	Corchorifatty acid F	Fatty Acyls	$C_{18}H_{32}O_5$	328.23	5.85	1.70	0.00027534	-1.7641	down
44	−	N-Propionylmethionine	Carboxylic acids and derivatives	$C_8H_{15}NO_3S$	205.08	3.31	1.57	0.00079991	-1.7002	down
